# New kid on the photosynthetic block: Ycf51 is a photosystem I assembly factor in cyanobacteria

**DOI:** 10.1093/plcell/koae014

**Published:** 2024-01-18

**Authors:** Guy Levin

**Affiliations:** Assistant Features Editor, The Plant Cell, American Society of Plant Biologists; Faculty of Biology, Technion, Haifa, 32000, Israel

During photosynthesis, pigments in light-harvesting complexes capture and transfer light energy to the reaction centers of the two chlorophyll-protein complexes of the electron transport chain: photosystem I (PSI) and II (PSII). Photosystems are constantly newly synthesized to accommodate growth or post-oxidative damage, and their assembly and integration to the thylakoid membrane are complicated processes, facilitated by numerous proteins. Although the assembly process of PSII has been extensively studied and many of its facilitators identified (Nickelsen and Rengstl 2013), experimental data describing the PSI assembly are lacking (Wittenberg et al. 2017). Hypothetical chloroplast open reading frames (*ycf*) are a group of conserved genes in cyanobacteria, algae, and plants that were discovered decades ago and encode proteins that were not associated with a known function (Hallick et al. 1993). Since their discovery, some Ycf proteins have been shown to have roles in the assembly of both photosystems, with Ycf3, Ycf4, and Ycf37 involved in PSI assembly (Rochaix 2011).

In this issue of *The Plant Cell*, **Guo-Zheng Dai, Wei-Yu Song, Hai-Feng Xu, and colleagues (Dai et al. 2024)** focus their work on unidentified *ycf* genes in cyanobacteria and discover that Ycf51 is a PSI assembly factor. The authors selected 12 *ycf* genes with an unknown function and generated knockout mutant strains of the cyanobacteria *Synechocystis* for each. Of those, the strain that was mutated in *ycf51 (ycf51*::C.K2) showed severe photoautotrophic growth retardation, suggesting a reduced capacity to perform photosynthesis (see Fig.). Consistent with this observation, chlorophyll fluorescence analysis revealed that the electron transport rate between PSII and PSI was severely reduced in *ycf51*::C.K2.

**Figure.**
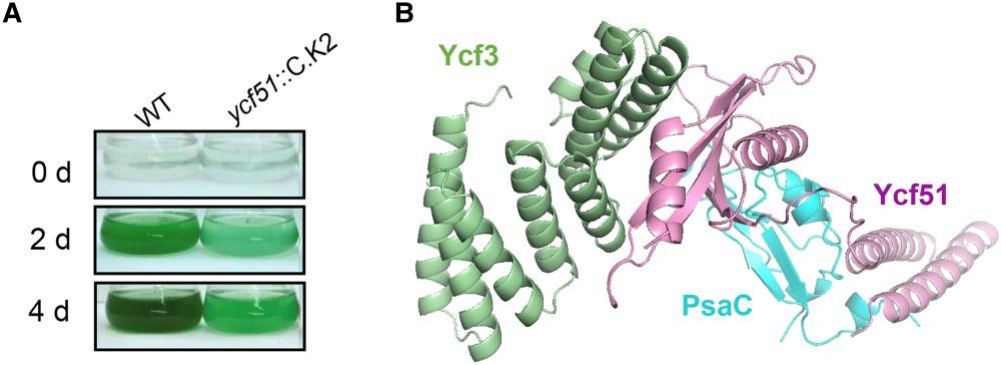
Ycf51 is a PSI assembly factor in cyanobacteria. **A)** disruption of *ycf51* in *Synechocystis* leads to retarded photoautotrophic growth. **B)** Simulated interaction model between Ycf 51, Ycf3, and PsaC. Adapted from Dai et al. (2024), Figures 1 and S8.

Further investigation analyzing P_700_ oxidation and low-temperature (77 K) fluorescence emission spectra showed that the PSI content is significantly reduced in *ycf51*::C.K2, leading to a shift from the optimal PSII:PSI ratio. Analyses of the PSI mRNA and protein content by RT-qPCR and immunoblots, respectively, show that the PSI content is regulated strictly at a post-translational level, suggesting Ycf51 may be important for PSI protein complex assembly. To explore this possibility, the wild-type *ycf51* gene fused to a glutathione-S-transferase (GST)-Tag was reintroduced to *ycf51*::C.K2 cells. Using anti-GST antibodies, Ycf51 was detected in the thylakoid membranes, where it may interact with other PSI proteins or accessory proteins required for PSI complex assembly. Indeed, in a series of GST and His-tag pull-down assays, combined with proteomics and yeast two-hybrid system analyses, Ycf51 was shown to interact with the known PSI assembly factor Ycf3 and the PSI subunit PsaC. To further elucidate this interaction, a structural model was simulated (see Fig.). Interestingly, disruption of *ycf51* did not impair the expression or localization of Ycf3, nor the stability of the PSI complex, suggesting that Ycf51 has an independent and direct role in the assembly of the PSI, but not in stabilizing the mature complex. Finally, BLAST analysis revealed that Ycf51 is conserved in all orders of cyanobacteria, but only in two lineages of photosynthetic eukaryotes, suggesting that Ycf51 is necessary for PSI assembly in cyanobacteria but was later replaced by other assembly factors in eukaryotes.

Here the authors have discovered the PSI assembly factor Ycf51 and provide insights into its mechanisms of action. Overall, Ycf51 interacts with PsaC and Ycf3 to facilitate PSI assembly, and the disruption of the *ycf51* gene leads to a major reduction in PSI content followed by a retarded photoautotrophic growth, highlighting its important role in PSI biogenesis (see Fig.). To further elucidate the role of Ycf51 in the PSI complex assembly, the authors now seek to identify Ycf51 in PSI intermediates from wild-type cells and determine the stage of assembly for which it is required. Considering that cyanobacteria were the earliest oxygenic photosynthetic organisms on Earth, it is also intriguing to reveal the roles of Ycf51 in the evolution of photosynthetic complexes during the transition from anoxic to oxic. Ycf51 may facilitate the protection of iron-sulfur clusters against oxygen damage as they become integrated into the PsaC subunit. A similar function was speculated for CGL71 in *Chlamydomonas reinhardtii* (Heinnickel et al. 2016; Nellaepalli et al. 2021).

## Dive Curated Terms

The following phenotypic, genotypic, and functional terms are of significance to the work described in this paper:

Ycf51 Gramene: Sll1702
Ycf51 Araport: Sll1702
